# Rapid detection method of soybean seed germination potential based on the PLSR-MLP fusion model

**DOI:** 10.3389/fpls.2025.1726266

**Published:** 2026-01-20

**Authors:** Shuo Liu, Zhengguang Chen

**Affiliations:** College of Information and Electrical Engineering, Heilongjiang Bayi Agricultural University, Daqing, China

**Keywords:** germination potential, model fusion, multilayer perceptron, near-infrared spectroscopy, partial least squares regression

## Abstract

In order to realize the rapid detection of soybean seed germination potential, this study designed a fusion model to solve the problem that the single model was insufficient in spectral feature analysis and the prediction performance was limited. The model combines the advantages of the Partial Least Squares Regression (PLSR) and the Multilayer Perceptron (MLP), and utilizing principal components extracted by PLSR as the input features for MLP to construct a soybean seed germination potential prediction model with both linear and nonlinear modeling capabilities. The PLSR module accurately extracts the linear features of the spectrum, and the MLP network further captures the nonlinear relationship between the spectral data and the target variable, which significantly improves the generalization ability of the model. The experimental results show that the prediction performance of the proposed PLSR-MLP fusion model (R_p_^2^ = 0.9534, RMSEP = 7.3821) is significantly improved compared with the single PLSR model (R_p_^2^ = 0.7284, RMSEP = 17.8154) and the single MLP model (R_p_^2^ = 0.7935, RMSEP = 15.5335). In the prediction of soybean germination potential, the PLSR-MLP model also outperforms other single models (Support Vector Machine, SVM; Random Forest, RF) and other fusion models such as PLSR-SVM and PLSR-RF. The PLSR-MLP fusion model effectively addresses the limitations of a single model’s performance enhancement potential and the susceptibility to overfitting. It provides a new method for the efficient evaluation of seed germination potential. It also has practical application value for precision seed selection in agriculture and offers a new idea for near-infrared spectrum modeling.

## Introduction

As a crucial crop for both grain and oil, soybeans are not only the primary source of high-quality plant protein and fat but also an essential and significant source of nutrients for people to balance their diet and maintain their health. Seed vigor, as the core element of the crop production system, is an important factor affecting seed germination efficiency and seedling growth quality. High-vigor seeds have the characteristics of a rapid germination rate, strong resistance to adversity, and large seedling biomass. By lowering the amount of seeding, they can guarantee the field emergence rate (Zhang and Fu, 2020), which will raise the yield.

In the seed quality evaluation system, germination rate and germination potential are the key indicators to characterize the biological characteristics of seeds. The former refers to the percentage of the number of normally germinated seeds and the tested seeds in the standard test cycle, reflecting the potential germination ability of the seeds. The latter describes the germination rate at the early stage of germination, usually the 3rd-5th day, reflecting the germination synchronicity and physiological activity intensity of the seeds. Studies have shown that seeds with high germination potential have better cell metabolic activity and antioxidant enzyme systems under the same germination rate. The field performance of seeds includes neat emergence and strong seedlings, which lays the foundation for constructing a high-yield crop population (Chen, 2012). It is worth noting that the decline rate of germination potential is significantly faster than the germination rate during seed aging. This characteristic makes the germination potential index more sensitive to reflect the changes in seed storage quality and becomes a reliable basis for evaluating the activity level of soybean seeds. Traditional seed vigor detection mainly depends on the artificial culture method and the biochemical labeling method. The former needs a 7-14-day germination cycle, which destroys the seed embryo structure. The latter relies on chemical reagents, which leads to irreversible damage to the sample.

The method for detecting seed vigor based on near-infrared spectroscopy technology has the characteristics of high efficiency and non-destructiveness and has been paid attention to by relevant scholars at home and abroad in recent years (Xia et al., 2024; Yuan et al., 2020). They have widely used this technology to detect crop seed activity and crop component content (Al-Amery et al., 2018; Kusumaningrum et al., 2018; Zhao et al., 2025; Li et al., 2018; Luo et al., 2017). The principle is that crops contain a large amount of water and organic matter, such as sugar, lipids, and proteins. By detecting the spectral absorption characteristics of the functional groups (N-H, O-H, C-H, etc.) of these substances, the composition of the crops can be quantitatively analyzed, and then the information related to seed activity can be obtained.

Most of the existing chemometric modeling methods based on near-infrared spectroscopy adopt linear regression modeling methods, such as the Partial Least Squares Regression (PLS) method. It has the characteristics of strong interpretability and high computational efficiency. It is widely used in the field of near-infrared spectroscopy detection, especially in the prediction of seed germination rate (Qu et al., 2019). The PLSR model can effectively characterize the spectral response by extracting latent variables, but its essence is a linear framework. The traditional linear model significantly limits its prediction accuracy when faced with the complex interaction effect between spectral data and seed vigor.

To break through the limitations of the linear model, researchers introduced nonlinear modeling methods (Xiao et al., 2023). For example, the Support Vector Machine (SVM) can effectively address the nonlinear relationship between spectral features through kernel function mapping (Bai et al., 2016; Peng et al., 2018). The Random Forest (RF) model reveals the nonlinear correlation between wavelength variables and seed vigor by using feature importance ranking (Du et al., 2024). In particular, deep learning models represented by Back Propagation (BP) neural networks have shown excellent nonlinear fitting ability in seed vigor detection (Yang et al., 2022). However, these methods face severe challenges in the scenario of small samples and high-dimensional spectral data. Too many hidden layer nodes of a neural network are prone to overfitting. The discretization of spectral continuous variables by decision tree models may cause information loss.

Most of the existing near-infrared spectral modeling methods adopt a single modeling strategy, which has several shortcomings. Firstly, a single model can only achieve limited performance. Second, linear models such as PLSR struggle to analyze data that exhibit complex nonlinear relationships. Third, models such as the BP neural network and Multilayer Perceptron (MLP) have strong nonlinear modeling capabilities, but in the face of small-sample, high-dimensional data, the model is prone to overfitting. Therefore, the single modeling method cannot give full play to the synergistic advantages of linear model feature decoupling and nonlinear model function approximation.

To address this challenge, scholars have begun to introduce model fusion strategies (Ge et al., 2019; Zhong et al., 2025; Jiang et al., 2024). The fusion model integrates the advantages of different algorithms, which becomes an effective way to improve the performance of the model (Shimim et al., 2025). Shi Rui et al. constructed an ECA-CNN fusion model to predict the vigor of wheat seeds, and the accuracy of the training set was as high as 99.17% (Shi et al., 2024). Yan Linyu et al. fused the CNN and LightGBM models for qualitative discrimination of rice seed vigor. The results indicated that the CNN-LightGBM fusion model could accurately identify the seed vigor levels of various rice varieties, demonstrating significantly better performance than a single model, along with good adaptability and stability (Yan et al., 2025). However, the above fusion models are all classification models for qualitative analysis and lack quantitative analysis of crop seed vigor. Additionally, the fusion method only combines similar types of models (nonlinear models) and does not effectively combine linear models with nonlinear models to take advantage of their different strengths.

In view of the above challenges and the actual needs for rapid detection of soybean seed germination potential, this study proposes a PLSR-MLP fusion model to realize the rapid detection of soybean seed germination potential. PLSR extracts the principal components of the spectrum, effectively eliminating collinearity and retaining key biochemical information. Combined with the nonlinear fitting ability of the MLP neural network, the accurate modeling of the complex mapping relationship between germination potential and spectral characteristics is realized. This method solves the problem that the performance improvement of a single linear model is limited, while a nonlinear model is easy to overfit and provides a new method for rapid and nondestructive detection of crop seed vigor.

## Materials and methods

### Material acquisition

The experimental materials were 14 main soybean varieties harvested in 2023 from Zhaoguang Farm (Mengdou 15, Dengke 15, Huinong 417, Huajiang 2, Heike 60, Heike 71, Heike 88, Heihe 43, Heihe 53, Soybean Longken 309, Soybean Longken 3002, Soybean Beidou 47, Jiadou 55, and Guangmin 5). There were 24 samples for each variety. Eight gradient samples of vigor levels were prepared by adjusting the aging time, and each level was set with three repetitions, forming a total of 14×8×3 = 336 experimental samples. In addition, the sample set also includes naturally aged seeds of two varieties, Heihe 53 and Dongqing 9 (storage period of 2 years), with 7 repeated samples for each variety. In summary, this study collected a total of 350 samples (336 + 14 = 350). The reference values for germination potential of all samples were obtained through the four-day germination rate testing method in accordance with the national standard GB/T 3543.4-1995 “Rules for Agricultural Seed Testing—Germination Test”.

### Artificial aging test

In this study, the high-temperature and high-humidity artificial accelerated aging method was used to obtain the soybean seed sample set with different vigor gradients, and the natural aging sample was used as a supplement. In this study, the intelligent incubator produced by Ningbo Saif Experimental Instrument Co., Ltd. was used as the artificial aging box for the high-temperature and high-humidity artificial aging test. Before the experiment, the surface of the incubator was sterilized with 75% ethanol solution. 130 seeds with grain fullness ≥95% and particle size variation coefficient <5% were selected from each variety as a sample, packed in 30 mesh nylon bags, and evenly spread on the incubator mesh frame. The accelerated aging parameters were set as constant temperature and humidity conditions with a temperature of 45 ± 1°C and relative humidity of 95% ± 1%. The aging gradient of 0-7d (0d, 1d, 2d, 3d, 4d, 5d, 6d, 7d) was established through time sequence control, in which the 0d group was used as the untreated control. After the treatment, the samples were naturally dried indoors (temperature 25 ± 2°C, humidity 40% ± 2%) for 3 days to reduce the seed moisture content to the level before aging.

### Near-infrared spectral acquisition

The spectra were collected using a Bruker TANGO Fourier transform near-infrared spectrometer (Germany). The spectral range was 11542–3946 cm^-^¹ (corresponding to the wavelength of 866–2534 nm), the resolution was 8 cm^-^¹, and each sample was scanned 16 times to take the average value as the spectrum of the sample.

### Germination potential determination

The soybean seed samples were placed in water at 25°C for 6 h. A layer of germination paper was placed in the germination box as a germination bed, and the seeds were evenly placed on the germination bed. Water was added until a thin layer of water film appeared on the germination paper, and then a layer of wet germination paper was covered over the seeds for germination culture. The number of seeds that met the germination standard was recorded daily; that is, the radicle was not spirally deformed and the plumule was more than half of the seed length. The moldy individuals were removed, and the evaporated water was replenished. At the end of the 7th day, the seed’s germination potential (germination rate on the 4th day) was obtained.

### Construction of the PLSR-MLP fusion model

PLSR is a multivariate statistical regression method for dealing with high-dimensional collinear data and is a classic spectral data modeling method. PLSR iteratively extracts a set of orthogonal latent variables (LVs) under the premise of retaining the maximum covariance between independent variables and dependent variables so as to realize data dimensionality reduction and feature extraction.

MLP is a typical feedforward neural network with strong nonlinear modeling ability and is one of the basic architectures of deep learning (Liu et al., 2022; Xie et al., 2022; Zhu et al., 2025). Its core structure consists of an input layer, a hidden layer, and an output layer. Through full connection (FC), layers transmit information layer by layer, and nonlinear activation functions extract and transform complex features.

**Figure 1:**
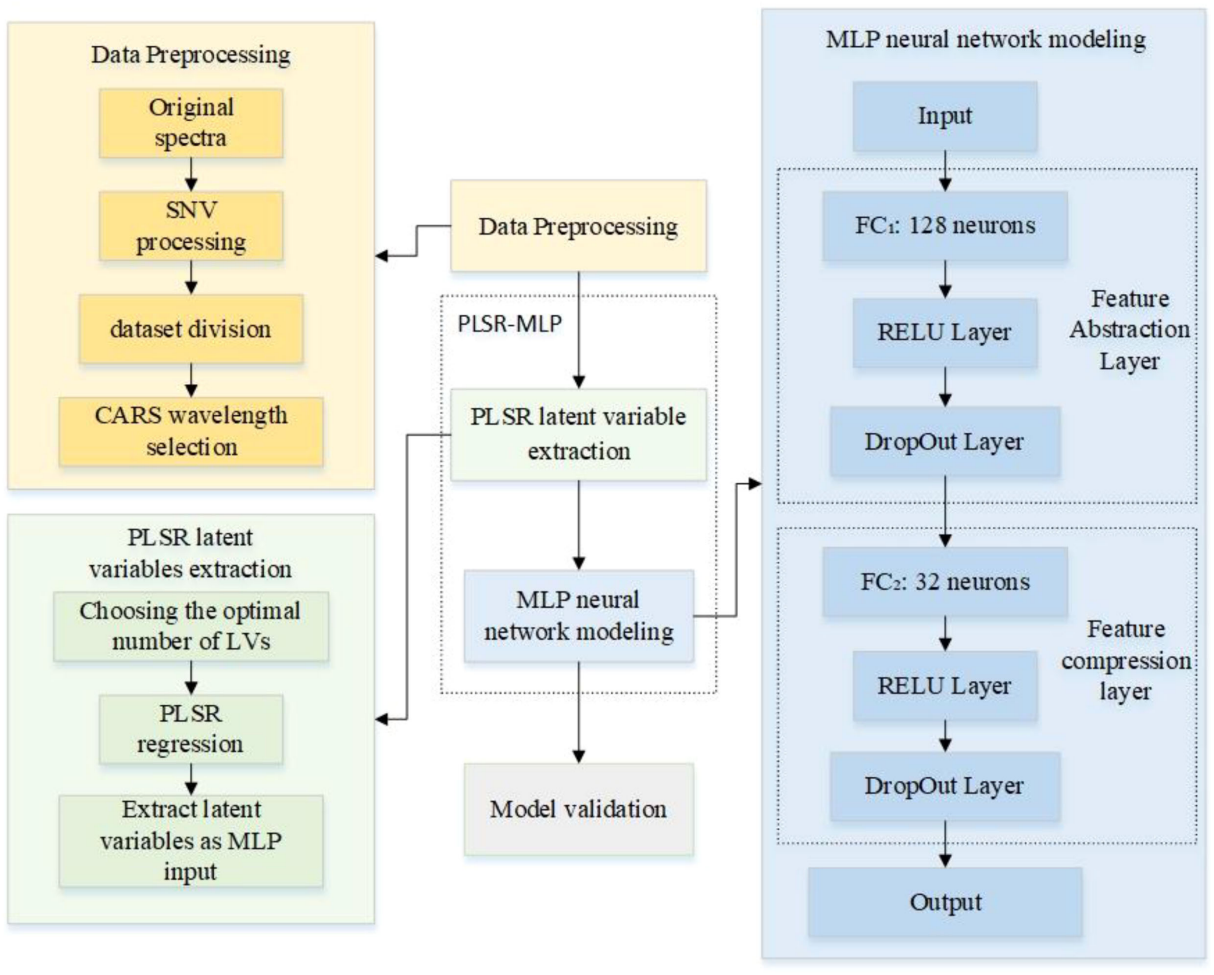
Structure of the PLSR-MLP fusion model.

The PLSR-MLP fusion model (**Figure 1**), constructed in this study, extracts key latent variables through the PLSR module to solve the problem of multi-collinearity in high-dimensional data. It provides low-noise feature input for the subsequent MLP network. The input layer of MLP receives the latent variables extracted by PLSR, and each input neuron corresponds to a feature dimension. The MLP includes two hidden layers, which are the feature abstraction layer and the feature compression layer. The feature abstraction layer is a fully connected layer (FC_1_) with 128 neurons, RELU activation, and a dropout that is used to randomly mask the output of some neurons. The purpose of this layer is to provide sufficient capacity for the network to learn more complex nonlinear relationships between input latent variables and between input latent variables and the target. The feature compression layer is a fully connected layer (FC_2_) with 32 neurons, RELU activation, and secondary dropout. The purpose of this layer is to compress the 128-dimensional abstract features of the previous layer into a low-dimensional representation space so that the network only retains the most critical information for the final prediction task and removes redundancy. The output layer is fully connected with one neuron.

The PLSR-MLP fusion model uses its powerful nonlinear modeling ability to adjust the linear features extracted by PLSR through adaptive weights. The RELU activation function is introduced into the MLP to correct the linear unit and realize the nonlinear transformation. At the same time, the dropout strategy is adopted to suppress the overfitting and enhance the generalization, and finally, the value of soybean germination potential is predicted through the output layer. The fusion model overcomes the limitations in expression ability of a single model and enhances the overall prediction accuracy by leveraging the synergy between both models. The structure of the PLSR-MLP fusion model is shown in **Figure 1**.

This study implemented data processing and modeling using the MATLAB R2021b platform. The training of the PLSR-MLP fusion model adopts the rmsprop optimizer. The maximum number of training rounds is 3500. The number of samples used in each iteration is 280, and the initial learning rate is set to 0.006.

### Symbol explanation

The article employs the coefficient of determination (R^2^), root mean square error (RMSE), and relative percent deviation (RPD) as model evaluation metrics. R_c_^2^ denotes the coefficient of determination for the modeling set, while R_p_^2^ denotes that for the prediction set. RMSECV is the root mean square error of cross-validation, RMSEC is the RMSE of the modeling set, and RMSEP is the RMSE of the prediction set. RPDc indicates the relative percent deviation of the modeling set, and RPDp indicates that of the prediction set.

## Results and discussion

### Near-infrared spectra of soybean seeds

**Figure 2:**
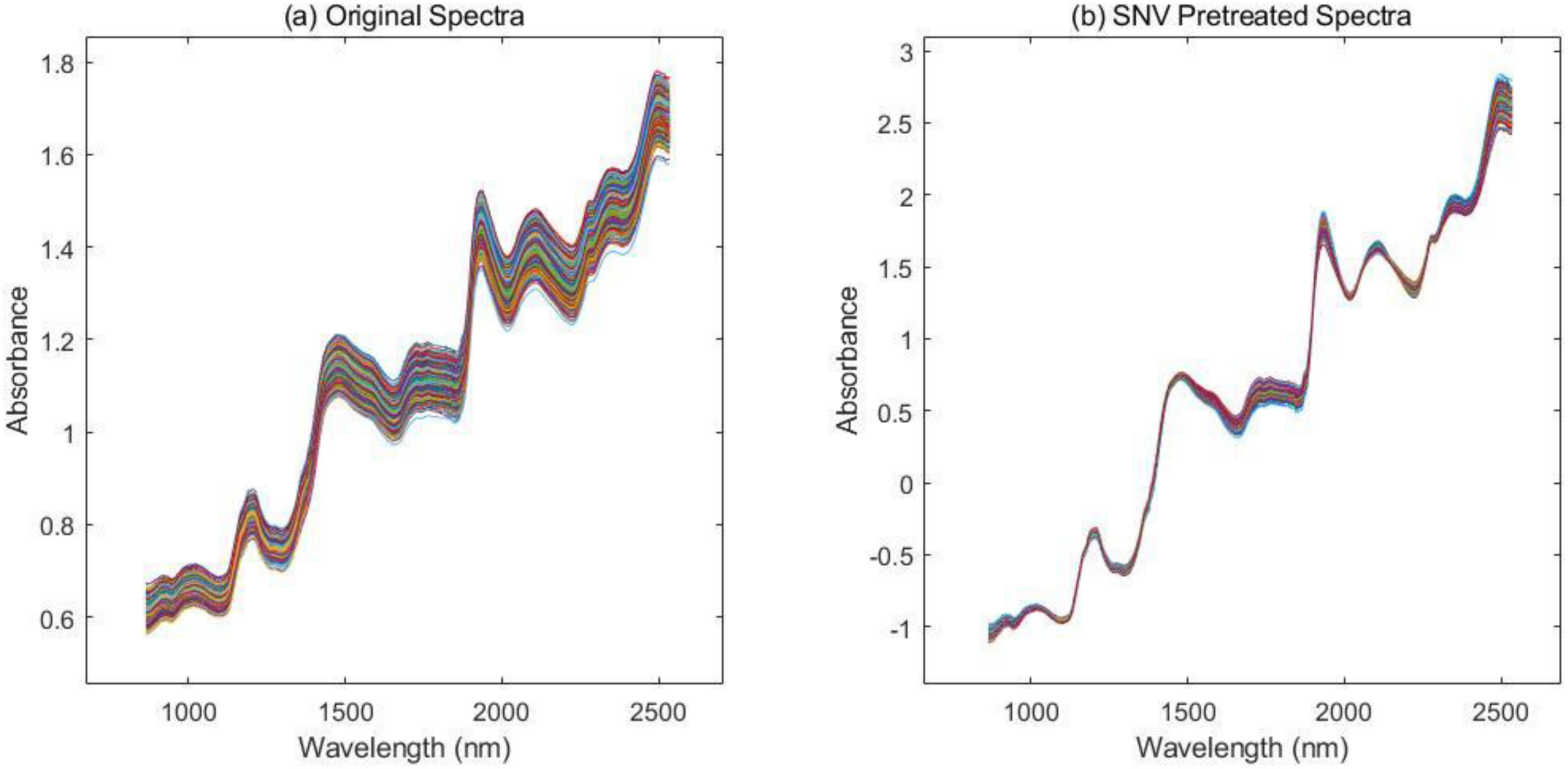
Comparison of near-infrared spectra for soybean seed samples **(A)** original spectra **(B)** SNV pretreated spectra.

The near-infrared diffuse reflectance spectra of 350 soybean samples are shown in (**Figure 2A**). There are obvious absorption peaks near 1200 nm, 1470 nm, 1950 nm, and 2300 nm. The characteristic bands of functional groups from various compounds overlap seriously, causing each prominent absorption peak to reflect contributions from multiple types of functional groups.

### Spectral preprocessing

Many factors, such as instrument noise and environmental noise, usually affect near-infrared spectral data. In this study, the standard normal variate (SNV) method was used to preprocess the spectral data. SNV is a preprocessing method based on the statistical characteristics of the spectrum itself, which is mainly used to eliminate the spectral baseline drift and amplification effects caused by the scattering of the sample surface, the size of the particles, or the difference in the optical path. Additionally, the SNV method exhibits strong resistance to outlier interference and is not sensitive to abnormal spectra or other characteristics. (**Figure 2B**) shows the soybean seed spectrum after SNV treatment. Compared with (**Figure 2A**), it can be seen that in the spectrum after SNV treatment, the spectral baseline is aligned, the amplitude is normalized, and the absorption peak related to the germination potential of soybean is effectively highlighted.

### Sample set partition

In this study, the SPXY (Sample set Partitioning based on joint X–Y distances) method was employed to divide the sample set (Galvao et al., 2005). The primary purpose of using SPXY is to ensure that the modeling set has a broader coverage than the prediction set in both the independent variable space (spectral features) and the dependent variable space (germination vigor), so that the model performs interpolation rather than extrapolation when applied to the prediction set. In contrast, random sample partitioning cannot effectively guarantee the representativeness of the modeling set with respect to the overall sample distribution. To evaluate the influence of different partitioning strategies, random splitting was also tested for comparison. The results indicated that the model exhibited evident overfitting on the prediction set, which may be attributed to the fact that some prediction samples fell outside the coverage of the modeling set. Therefore, the SPXY method was ultimately adopted to partition the sample set in order to improve the stability and predictive reliability of the model. The whole sample set was divided into a modeling set (280 samples) and an independent prediction set (70 samples) at a ratio of 8:2. The division results are shown in **Table 1**.

**Table 1:** Soybean germination potential dataset division results.

Sample type	Sample size	Minimum value(%)	Maximum value(%)	Average value(%)	Standard deviation(%)	Coefficient of variation
Overall sample	350	0.00	100.00	68.4371	31.0722	0.4540
Modeling Set	280	0.00	100.00	67.0929	30.3064	0.4517
Prediction Set	70	0.00	100.00	73.8143	33.6578	0.4560

### Wavelength selection

**Figure 3:**
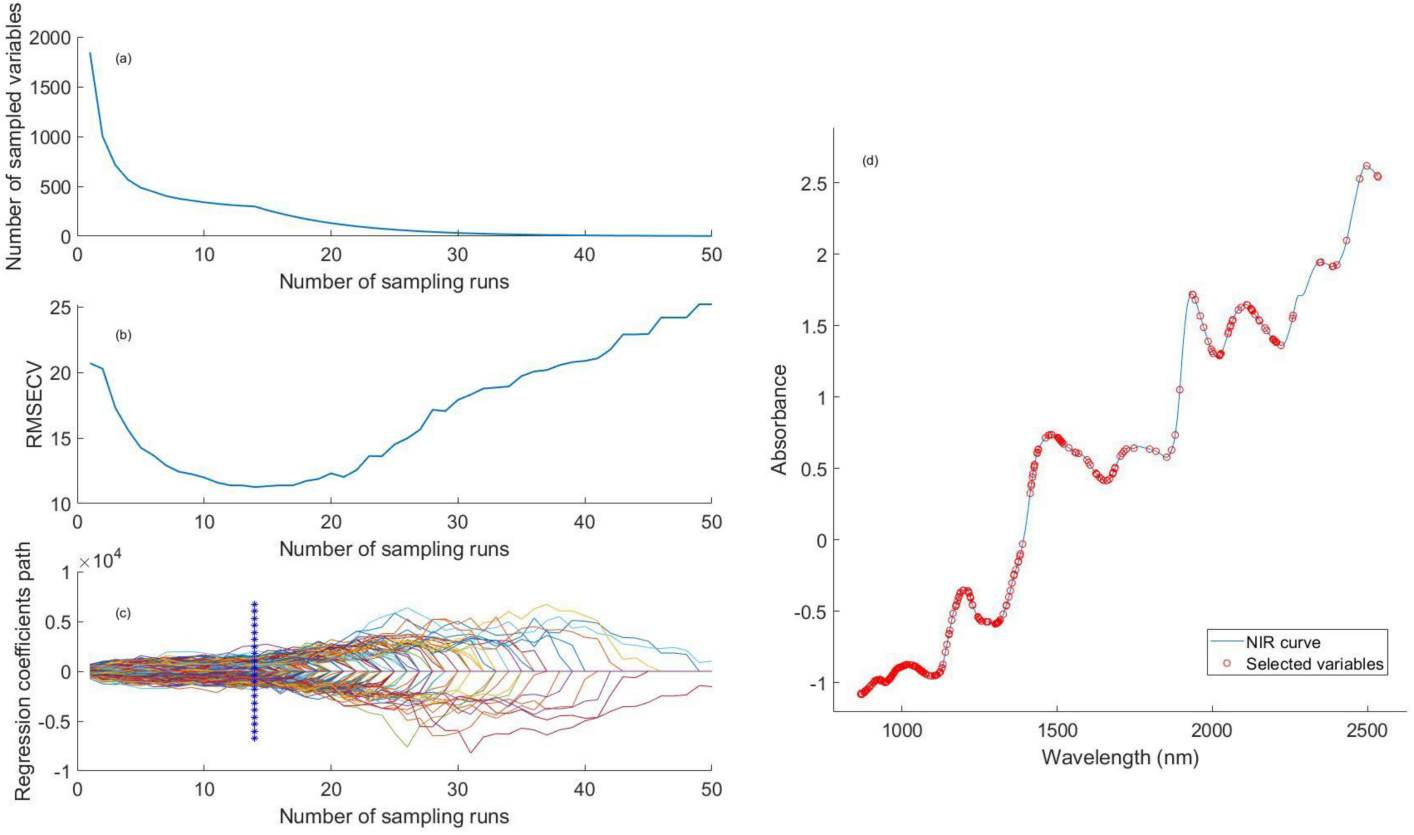
CARS wavelength selection **(A)** Number of Selected Wavelengths **(B)** RMSECV Optimization Process **(C)** Regression Coefficient Path **(D)** wavelength selection results.

In this study, the competitive adaptive reweighted sampling (CARS) algorithm was used to select the characteristic wavelengths of the near-infrared spectral data (Li et al., 2009). After multiple rounds of iterative optimization, the original 1,845 spectral wavelength variables were compressed to 302 characteristic wavelength variables, which effectively eliminated the problem of high collinearity and the interference of non-information noise. **Figure 3** shows the CARS wavelength selection process based on 10-fold cross-validation, where the horizontal axis represents the number of iterations (1~50 times). The vertical axis of (**Figure 3A**) is the number of selected wavelengths. (**Figure 3B**) shows that the minimum cross-validation root mean square error (RMSECV) is obtained at the 14th iteration, and the corresponding 302 characteristic wavelengths are determined as the optimal variable subset. (**Figure 3C**) shows the regression coefficient path. (**Figure 3D**) shows the location of the selected characteristic wavelengths.

In the 900–1400 nm spectral region, the third overtone vibration of CH_2_/CH_3_ groups in soybean seed oil and the vibration of C-O/C-H bonds in starch form a strong absorption superposition. The oil content determines the energy metabolism efficiency in the early stage of seed germination, and the soluble sugar produced by starch decomposition directly affects the germination trend of soybean seeds. In the 1450–1700 nm band, the first overtone absorption of the protein N-H bond and the first overtone absorption of the water O-H bond are covered. In the 2000–2400 nm spectral region, the combination frequency absorption peak of the water molecule O-H bond generally occurs in this band. The combination frequency vibration of the protein N-H bond generally occurs at 2050–2200 nm. The first overtone vibration of the oil C-H bond generally occurs at 2250–2350 nm, providing energy support for seed germination (Silva et al., 2024). The above screening results of characteristic wavelengths show that the CARS algorithm accurately captures the chemical component information closely related to seed germination and lays a data foundation for establishing a high-robustness germination potential prediction model.

### PLSR-MLP modeling results

PLSR models usually use cross-validation to determine the optimal number of latent variables (LVs). This study used 10-fold cross-validation to analyze the data set. The results demonstrated that the PLSR model achieved a minimum RMSECV value of 18.8691 with 11 LVs. However, the preliminary experiment found that when the LVs were 11, the coefficient of determination R_c_^2^ of the PLSR-MLP fusion model on the test set was 0.8675; opportunities for enhancement remain. The use of 11 LVs makes it challenging to fully characterize the intricate link between spectral data and concentration variables. To evaluate the influence of the number of LVs on the model performance more comprehensively, this study adopts the variance contribution rate analysis method to investigate the information interpretation ability of the spectral matrix (X) and the response variable (Y) under different numbers of LVs. This method can not only avoid the local optimal problem that may be caused by cross-validation but also provide a more reliable basis for the selection of PLSR-MLP model complexity from the perspective of data interpretation.

**Figure 4:**
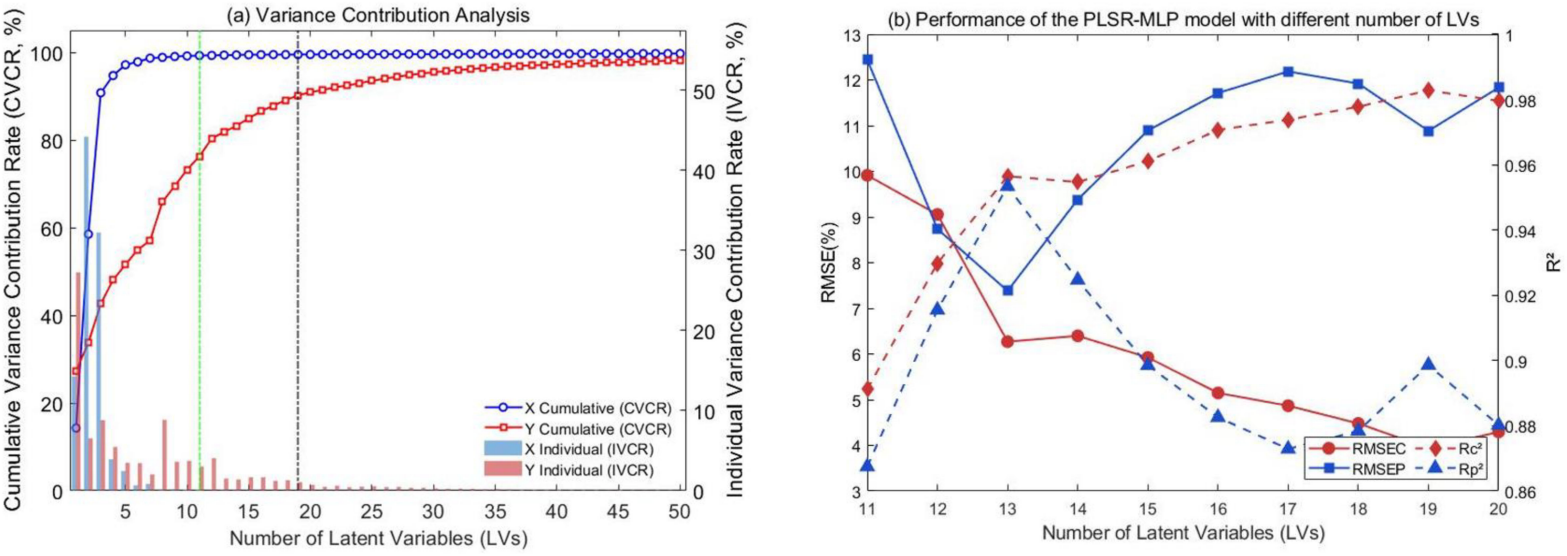
Variance contribution and model performance with different LVs **(A)** Variance contribution rate analysis **(B)** Performance of the PLSR-MLP model with different number of LVs.

The number of LVs is determined by assessing the explanatory power of different numbers of LVs for the spectral matrix (X) and the response variable (Y). The specific method is to calculate two key indicators: Individual Variance Contribution Rate (IVCR) and Cumulative Variance Contribution Rate (CVCR). (**Figure 4A**) shows the change in the variance contribution rate of X and Y when the number of LVs increases from 1 to 50. As can be seen from (**Figure 4A**), with the increase in the number of LVs, the CVCR of X quickly approached 100%, while the CVCR of Y increased relatively slowly. When the number of LVs increased to 11, the CVCR of X reached 99.2657%, indicating that the first 11 LVs could explain more than 99.2657% of the information of the spectral data. However, the CVCR of Y was only 76.2643% at this time, indicating that the coverage rate of the prediction information of the 11 LVs on the germination potential of soybean seeds still had significant room for improvement. It is worth noting that the CVCR of Y reaches 90.1755% (>90%) when the number of LVs increases to 19. In particular, the IVCR values for the 20th LV concerning X and Y were only 0.0136% and 0.7231% (both <1%), respectively, indicating that their contributions were negligible. Therefore, continuing to increase the number of LVs contributes very little to the improvement of the CVCR and IVCR of X or Y.

Based on the above analysis, this study used 11 to 20 LVs as the input of MLP, respectively, and obtained the performance parameters of the PLSR-MLP model under different LV numbers (**Table 2**). (**Figure 4B**) shows the effect of the change in the number of LVs on the performance of the PLSR-MLP fusion model. In (**Figure 4B**), the horizontal axis represents the number of LVs, the left vertical axis represents the RMSE value, and the right vertical axis represents the R^2^ value. (**Figure 4B**) illustrates that the number of LVs is 13, a pivotal node. At this time, the RMSEP of the test set attains its minimum value, whereas R_P_^2^ achieves its maximum. When the number of LVs surpasses 13, the RMSEP of prediction escalates significantly. Although there is a slight decrease when LVs = 19, it increases significantly when LVs = 20. In the training set, the RMSEC did not reach its lowest point at LVs = 13, but its decline remained limited as the number of LVs increased. The trend of R² for the model on both the training set and the test set indicated that the model exhibited an overfitting trend when the LVs exceeded 13.

**Table 2:** Prediction results of the PLSR-MLP model with different number of LVs.

Number of LVs	R_c_^2^	RMSEC	RPDc	R_p_^2^	RMSEP	RPDp
11	0.8912	9.9132	3.0370	0.8675	12.4437	2.7670
12	0.9298	9.0549	3.8026	0.9155	8.7381	3.4455
13	0.9565	6.2706	4.8013	0.9534	7.3821	4.6643
14	0.9547	6.3980	4.7057	0.9247	9.3818	3.6701
15	0.9611	5.9246	5.0817	0.8985	10.8937	3.1608
16	0.9707	5.1473	5.8490	0.8826	11.7140	2.9394
17	0.9738	4.8677	6.1850	0.8729	12.1866	2.8254
18	0.9778	4.4799	6.7204	0.8784	11.9209	2.8884
19	0.9828	3.9411	7.6392	0.8986	10.8864	3.1629
20	0.9796	4.2937	7.0118	0.8802	11.8321	2.9101

Therefore, the number of LVs was finally selected as 13, and the 13-dimensional latent variables were used as the input of MLP for the prediction of soybean germination potential. This selection effectively avoided the risk of overfitting while ensuring that the model had sufficient prediction accuracy (Y matrix CVCR > 90%). Through this optimization process, the 302-dimensional high-dimensional spectral data was successfully reduced to 13-dimensional key features, which not only significantly improved the computational efficiency but also achieved the dual optimization goals of data denoising and feature enhancement.

### Comparison with single model

To highlight the advantages of the fusion model, we established a PLSR model and an MLP model, respectively. The optimal number of latent variables for the single PLSR model was determined to be 11 by 10-fold cross-validation. The input of the single MLP model was 302-dimensional spectral data, and its hidden layer structure was consistent with the MLP part in the PLSR-MLP model.

The prediction results are shown in **Table 3**. The performance of the PLSR-MLP fusion model on the test set is better than that of the single model. Although the R_c_^2^ of the MLP model on the training set is better than that of the PLSR-MLP fusion model, the MLP model has an obvious overfitting phenomenon. On the test set, the coefficient of determination (R_p_^2^ = 0.9534) of the PLSR-MLP fusion model was 30.89% and 20.15% higher than that of the single PLSR model (R_p_^2^ = 0.7284) and the single MLP model (R_p_^2^ = 0.7935), respectively. At the same time, its root mean square error (RMSEP = 7.3821) was 58.56% and 52.48% lower than that of the single PLSR model (RMSEP = 17.8154) and the single MLP model (RMSEP = 15.5335), respectively. In addition, the RPDp value of the PLSR-MLP fusion model is as high as 4.6643, which is greater than 3, further confirming its excellent prediction ability (Zhao et al., 2025).

**Table 3:** Comparative prediction performance of the PLSR-MLP fusion model versus other models.

Modeling method	R_c_^2^	RMSEC	RPDc	R_p_^2^	RMSEP	RPDp
PLSR	0.8307	12.3649	2.4349	0.7284	17.8154	1.9327
MLP	0.9694	5.2537	5.7306	0.7935	15.5335	2.2166
PLSR-MLP	0.9565	6.2706	4.8013	0.9534	7.3821	4.6643
SVM	0.7986	13.4859	2.2325	0.7497	17.1024	2.0133
RF	0.7661	14.6297	2.0716	0.6311	20.2971	1.6583
PLSR-SVM	0.9035	9.3352	3.2251	0.8743	12.1209	2.8407
PLSR-RF	0.7889	13.8076	2.1805	0.4283	25.8478	1.3321

(**Figures 5A–C**) show the prediction results of the single PLSR model, the single MLP model, and the PLSR-MLP fusion model on the data set, respectively. The prediction effect of the single PLSR model is not satisfactory on both the training set and the test set. This is because PLSR, a linear regression model, finds the direction of maximum covariance between the independent variable and the dependent variable (Qu et al., 2019). However, the germination process of soybean seeds involves complex physiological and biochemical mechanisms, which are affected by the interaction of multiple factors, and there is a strong nonlinear relationship between its spectral data and germination potential. As a linear model, PLSR cannot effectively capture these complex nonlinear patterns, resulting in limited prediction ability. The single MLP model shows an obvious overfitting phenomenon. This is mainly because the single MLP directly receives the 302-dimensional spectral features, which contain substantial noise and redundant variations unrelated to germination potential. Owing to its strong nonlinear fitting ability, the MLP tends to learn the noise, random fluctuations, and specific samples details in the training data rather than the general generalization rules (Zhang et al., 2023). At the same time, the relatively limited amount of training data exacerbates this phenomenon, resulting in favorable performance on the training set but poor prediction results on the test set with different noise patterns.

**Figure 5:**
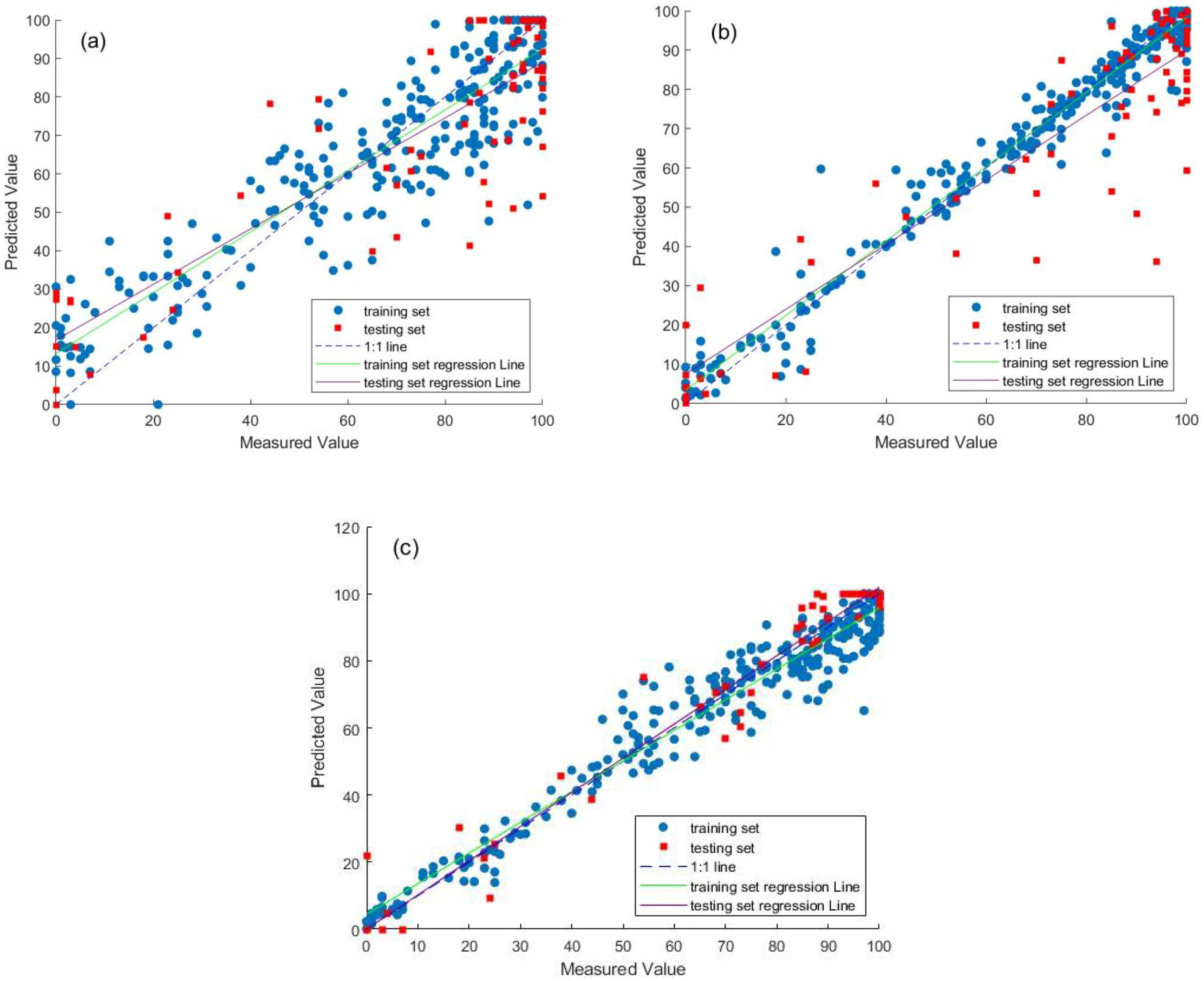
Comparison of prediction results for different models **(A)** PLSR model **(B)** MLP model **(C)** PLSR-MLP model.

### Comparison with other models

To further evaluate the performance of the PLSR-MLP fusion model, this study selected SVM and RF, which are widely used in the field of machine learning, as benchmark models for comparative analysis. These two algorithms have a solid theoretical foundation and strong universality and are typical representatives in the field of spectral analysis and agricultural phenotype prediction (Ding et al., 2023; Li et al., 2015; Zhang et al., 2024).

SVM is a supervised learning algorithm based on statistical learning theory. By mapping data to high-dimensional space through a kernel function to construct the optimal hyperplane, it can effectively address spectral features and maintain excellent generalization ability under limited sample conditions (Li et al., 2025). In this study, the Gaussian kernel function and 10-fold cross-validation were used. The optimal parameters of the SVM model for predicting soybean germination potential were determined by the grid optimization method. The regularization parameter C was 5, and the ϵ-insensitive region was 0.05. The RF model generates multiple sub-datasets through bootstrap resampling. For each subset, an unpruned regression tree is constructed. A random subset of features is selected when the node is split, and the average of all decision tree predictions is output (Jing et al., 2025). In this study, the optimal parameters of the RF model for predicting soybean germination potential were determined by Bayesian optimization (Cui and Yang, 2018). The parameters numTrees, max_features, and minLeafSize were 260, 17, and 5, respectively.

As shown in **Table 3**, the R_c_^2^ of the SVM model in the training set of soybean germination potential was 0.7986, and the RMSEC was 13.4859. The R_p_^2^ of the test set was 0.7497, and the RMSEP was 17.1024. The R_c_^2^ of the RF model in the training set was 0.7661, and the RMSEC was 14.6297. The R_p_^2^ of the test set was 0.6311, and the RMSEP was 20.2971. In contrast, the prediction performance of the PLSR-MLP fusion model on the test set was significantly better than that of the benchmark model. Its R_p_^2^ was 27.17% and 51.07% higher than that of the SVM model and the RF model, respectively. At the same time, its RMSEP was 56.84% and 63.63% lower than that of the SVM model and the RF model, respectively.

The above results indicate that the prediction performance of SVM and RF models on soybean germination potential is not ideal. The reason may be that the SVM model can deal with nonlinear relationship data, but it is extremely sensitive to noise when searching for the optimal separation hyperplane in high-dimensional space (Cruz et al., 2021). Soybean seed spectra and germination potential data are easily affected by uncontrollable factors such as micro-environmental differences and individual seed differences (Li et al., 2025). Even after SVN preprocessing and CARS screening, certain random noise remains in the 302-dimensional spectra. In addition, the 302-dimensional features are too high relative to the limited sample size (Cervantes et al., 2020), resulting in a decline in the performance of the SVM-based prediction model. The RF model introduces randomness, and there will be some prediction errors when evaluating the target variable (Jiang et al., 2024). Simultaneously, feature redundancy and noise readily impact the RF model (Kong et al., 2025). In addition, the sample size of this study is small, and it is difficult to construct enough and diverse decision trees to fully capture the complex relationship of the data, resulting in insufficient model stability and reduced generalization ability (Sun et al., 2023). Therefore, the prediction errors of the training set and the test set are both large.

To further explore the performance of the fusion model, this study further fused PLSR with SVM and RF, respectively, to construct PLSR-SVM and PLSR-RF models. It should be noted that the PLSR structure of these two fusion models is consistent with the PLSR-MLP model, and both use 13-dimensional latent variables as the input of the subsequent model. The experimental parameters of the PLSR-SVM and PLSR-RF models are the same as those of the single SVM and single RF models. The performance of the two fusion models is detailed in **Table 3**.

The PLSR-SVM model performed well on the training set (RMSEC = 9.3352, R_c_^2^ = 0.9035), and its performance on the test set (RMSEP = 12.1209, R_p_^2^ = 0.8743) was also greatly improved compared with the single SVM model. This further illustrates that the process of introducing PLSR to extract latent variables in the fusion model can effectively filter noise, enhance the ability of feature representation, and thus improve the overall performance of the model. However, the performance of PLSR-SVM is not as effective as that of PLSR-MLP, which may be because the latent variables extracted by PLSR retain the complex nonlinear structure in the original data, although they are reduced in dimension and denoised. MLP can learn this structure more fully with its deep nonlinear transformation ability (Zhu et al., 2025).

The performance of the PLSR-RF model was different. The results on the training set were RMSEC = 13.8076 and R_c_^2^ = 0.7889. The results on the test set were RMSEP = 25.8478 and R_p_^2^ = 0.4283. Although the R_c_^2^ of the training set is better than that of the single RF model, the R_p_^2^ of the test set is significantly reduced. This phenomenon indicates that the model has the problem of insufficient generalization ability. The reason may be that when the RF model inputs the 13-dimensional latent variables after PLSR dimensionality reduction instead of the original high-dimensional features, the RF may be more likely to capture the specific patterns in the training data, resulting in poor performance on the test set. In addition, although the latent variables extracted by PLSR remove some noise, they may also lose some original feature information that is critical to the RF decision tree splitting, thereby weakening the predictive ability of the RF model.

In summary, the PLSR-MLP fusion model constructed in this study effectively overcomes the challenges of high-dimensional noise, small samples, and model parameter optimization by integrating the dimensionality reduction and denoising capabilities of PLSR with the powerful adaptive learning capabilities and deep nonlinear modeling advantages of MLP (Zhang et al., 2023), thereby achieving better prediction performance.

## Conclusion

This work successfully developed a PLSR-MLP fusion model that accurately predicts soybean seed germination potential via near-infrared spectral prediction. The method effectively overcomes the limitations of a singular model in spectral analysis, such as feature redundancy and nonlinear relationship modeling, using a dual-model fusion mechanism. First, the PLSR algorithm is used to compress the features of the original spectrum, and the key principal component variables closely related to the germination potential are extracted while reducing the data dimension. And then, the powerful nonlinear mapping ability of the MLP network is used to construct a deep feature interaction network, which significantly improves the model’s ability to represent complex relationships. The experimental results indicate that the prediction accuracy R_p_^2^ of the model reaches 0.9534. This two-stage fusion modeling strategy not only provides agricultural workers with a rapid and non-destructive seed vigor assessment tool, but more importantly, it offers an efficient technical means for quality screening before soybean sowing and precise management of germplasm resource banks. Although this study provides a new idea for multi-model fusion modeling, a thorough evaluation of the performance of the three fusion models (PLSR-MLP, PLSR-SVM, and PLSR-RF) reveals that not any fusion model can improve the model prediction performance. The effectiveness of the model fusion strategy must be validated via experimentation for a particular data set. Future research will explore the combination of transfer learning and data enhancement technology to solve the problem of improving the generalization ability of the model under small samples, and at the same time, it is planned to extend this framework to a universal detection system for multiple crop varieties.

## Data Availability

The raw data supporting the conclusions of this article will be made available by the authors, without undue reservation.

## References

[B1] Al-Amery M. Geneve R. L. Sanches M. F. Armstrong P. R. Maghirang E. B. Lee C. . (2018). Near-infrared spectroscopy used to predict soybean seed germination and vigour. Seed Sci. Res. 28, 245–252. doi: 10.1017/s0960258518000119

[B2] Bai J. Peng Y.-K. Wang W.-X. (2016). Discrimination of vitality of maize seeds based on near visible infrared spectroscopy. J. Food Saf. Qual. 7, 4472–4477. doi: 10.19812/j.cnki.jfsq11-5956/ts.2016.11.043

[B3] Cervantes J. Garcia-Lamont F. Rodríguez-Mazahua L. Lopez A. (2020). A comprehensive survey on support vector machine classification: Applications, challenges and trends. Neurocomputing 408, 189–215. doi: 10.1016/j.neucom.2019.10.118

[B4] Chen D.-H. (2012). Discussion on the importance of seed germination potential to field emergence rate of crops. China Seed Industry 03), 49–50. doi: 10.19462/j.cnki.1671-895x.2012.03.025

[B5] Cruz S. Guerra R. Brazio A. Cavaco A. M. Antunes D. Passos D. (2021). Nondestructive simultaneous prediction of internal browning disorder and quality attributes in ‘Rocha’ pear (Pyrus communis L.) using VIS-NIR spectroscopy. Postharvest Biol. Technol. 179. doi: 10.1016/j.postharvbio.2021.111562

[B6] Cui J.-X. Yang B. (2018). Survey on bayesian optimization methodology and applications. J. Software 29, 3068–3090. doi: 10.13328/j.cnki.jos.005607

[B7] Ding Z.-Y. Yue X.-J. Zeng F.-G. Shi H.-W. Peng W. Xiao J.-Y. (2023). Spectral detection of maize seed vigor based on machine learning and deep learning. J. Huazhong Agric. Univ. 42, 230–240. doi: 10.13300/j.cnki.hnlkxb.2023.03.027

[B8] Du W.-L. Guo P. Liu X. (2024). Detection of delinted cotton seed vigor based on hyperspectral wavelet features. Trans. Chin. Soc. Agric. Eng. 40, 174–186.

[B9] Galvao R. K. Araujo M. C. Jose G. E. Pontes M. J. Silva E. C. Saldanha T. C. (2005). A method for calibration and validation subset partitioning. Talanta 67, 736–740. doi: 10.1016/j.talanta.2005.03.025, PMID: 18970233

[B10] Ge X. Sun J. Lu B. Chen Q. Xun W. Jin Y. (2019). Classification of oolong tea varieties based on hyperspectral imaging technology and BOSS-LightGBM model. J. Food Process Eng. 42. doi: 10.1111/jfpe.13289

[B11] Jiang X.-G. He C. Jiang N. Li L.-S. Zhu M.-W. Liu Y.-D. (2024). Discrimination of apple origin and prediction of SSC based on multi-model decision fusion. Spectrosc. Spectral Anal. 44, 2812–2818.

[B12] Jing X. Ye Q.-X. Li B.-Y. Zhang Z.-H. Zhao T.-H. (2025). Remote sensing monitoring of wheat stripe rust based on shape characteristics of full-spectrum SIF. Trans. Chin. Soc. Agric. Machinery 56, 468–476.

[B13] Kong K.-F. Chen Y.-L. Chen F. Deng Z.-X. Wang Z. Xiao Y.-J. (2025). Multi-model decision level fusion of soil slope stability predictive model and interpretability analysis. J. Railway Sci. Eng., 1–13. doi: 10.19713/j.cnki.43-1423/u.T20250822

[B14] Kusumaningrum D. Lee H. Lohumi S. Mo C. Kim M. S. Cho B. K. (2018). Non-destructive technique for determining the viability of soybean (Glycine max) seeds using FT-NIR spectroscopy. J. Sci. Food Agric. 98, 1734–1742. doi: 10.1002/jsfa.8646, PMID: 28858390

[B15] Li W.-J. Lei R. Zeng Y. Wang Z.-X. (2025). Construction of seed vigour prediction models for different seed coat soybean based on near-infrared spectroscopic techniques. Chin. J. Oil Crop Sci., 1–14. doi: 10.19802/j.issn.1007-9084.2024371

[B16] Li W. Li Y. Li G.-K. Gao L. Chen M.-Z. Lu J.-G. . (2018). Seed vigor detection of sweet corn by near infrared spectroscopy under high temperature stress. J. Nucl. Agric. Sci. 32, 1611–1618.

[B17] Li H. Liang Y. Xu Q. Cao D. (2009). Key wavelengths screening using competitive adaptive reweighted sampling method for multivariate calibration. Anal. Chim. Acta 648, 77–84. doi: 10.1016/j.aca.2009.06.046, PMID: 19616692

[B18] Li H.-H. Lu W. Du C.-W. Ma F. Luo H. (2015). Study on rapid and non-destructive detection of rice seed vigor based on photoacoustic spectroscopy combined with LS-SVR. Chin. J. Of Lasers 42, 280–289.

[B19] Liu Y. Liu F.-L. Wu J.-W. Li K.-S. Wang R.-W. (2022). Keratoconus model for auxiliary diagnosis based on MLP neural network. J. Optoelectronics·Laser 33, 1201–1206. doi: 10.16136/j.joel.2022.11.0128

[B20] Luo L.-P. Liu X.-X. Yin Q. Du S.-G. Huang X.-Y. Luo H.-L. (2017). Application of near infrared spectroscopy in seed vigor determination for Brassica plants. J. Nanchang Univ. (Natural Science) 41, 66–71. doi: 10.13764/j.cnki.ncdl.2017.01.014

[B21] Peng Y.-K. Zhao F. Li L. Xing Y.-Y. Fang X.-Q. (2018). Discrimination of heat-damaged tomato seeds based on near infrared spectroscopy and PCA-SVM method. Trans. Chin. Soc. Agric. Eng. 34, 159–165.

[B22] Qu G. Chen Z.-G. Zhang Q.-H. (2019). Study on germination rate of rice seed based on uninformation variable elimination method. Jiangsu J. Agric. Sci. 35, 1015–1020.

[B23] Shi R. Zhang H. Wang C. Kang K. Luo B. (2024). Detection of wheat single seed vigor using hyperspectral imaging and spectrumFusion strategy. Spectrosc. Spectral Anal. 44, 3206–3212.

[B24] Shimim F. N. Fortin M. P. Nocco M. Whitaker B. Gal A. Diaz D. . (2025). Data fusion approach for predicting high resolution estimates of crop evapotranspiration. Precis. Agric. 26. doi: 10.1007/s11119-025-10273-x

[B25] Silva M. F. Roque J. V. Soares J. M. Moura L. Medeiros A. Silva F. . (2024). Near infrared spectroscopy for the classification of vigor level of soybean seed. Rev. Ciec. Agron. 55. doi: 10.5935/1806-6690.20240005

[B26] Sun H.-B. Yang J.-G. Jin H.-W. Tu H.-B. Zhou X.-L. Zhao H. (2023). NOx prediction model for coal-fired boiler based on bayesian optimization-random forest regression. J. Chin. Soc. Power Eng. 43, 910–916. doi: 10.19805/j.cnki.jcspe.2023.07.013

[B27] Xia X.-Y. Li F. Xie K. Shao M.-X. Wang X.-J. Ma G.-C. . (2024). Research progress in non-destructiveTesting technology for crop seed viability. Seed 43, 64–73. doi: 10.16590/j.cnki.1001-4705.2024.10.064

[B28] Xiao H. Chen Z. Yi S. Liu J. (2023). Rapid detection of maize seed germination rate based on Gaussian process regression with selection kernel function. Vibrational Spectrosc. 129. doi: 10.1016/j.vibspec.2023.103595

[B29] Xie S.-F. Zeng Y. Zhang J.-H. Zhang Y.-B. Xiong S. (2022). Atmospheric weighted mean temperature model based on MLP neural network. J. Geodesy Geodynamics 42, 1105–1110. doi: 10.14075/j.jgg.2022.11.002

[B30] Yan L.-Y. Cao J.-W. Yang Y.-Q. Liu Y.-J. Zou Y. Cheng C.-X. . (2025). Quantitative detection of vigor of rice seeds based on hyperspectral imaging and integrated model. Laser Optoelectronics Prog., 1–15.

[B31] Yang D.-F. Li A.-C. Liu J.-M. Chen Z. G. Shi C. Hu J. (2022). Optimization of seed vigor near-infrared detection by coupling mean impact value with successive projection algorithm. Spectrosc. Spectral Anal. 42, 3135–3142.

[B32] Yuan J. Zheng W. Qi H.-N. Gao L. Hu X.-J. Zhao G.-W. . (2020). Progress in research of optical non-destructive test technology for seed vigor. Crops 05), 9–16. doi: 10.16035/j.issn.1001-7283.2020.05.002

[B33] Zhang C.-X. Fu Z. (2020). Analysis and countermeasure of production dilemma of chinese soybean under the international background. J. Hebei Univ. Econ Business (Comprehensive Edition) 20, 73–78. doi: 10.14178/j.cnki.issn1673-1573.2020.04.012

[B34] Zhang H.-L. Nie X. Liao S.-M. Zhan B.-S. Luo W. Liu S.-L. . (2024). Feasibility study on identification of seeds of hong kong seeds 49, october red and september fresh cabbage based on visible/shortwave near-infrared spectroscopy of partial least squares discriminant (PLS-DA) and least squares support vector machine (LS-SVM). Spectrosc. Spectral Anal. 44, 1718–1723.

[B35] Zhang C. Zhu Y.-J. Feng G.-H. (2023). Research on storage quality detection method of blueberry based on ensemble learning and near-infrared spectroscopy. Food Fermentation Industries 49, 306–314. doi: 10.13995/j.cnki.11-1802/ts.035198

[B36] Zhao J.-Y. Chen Z.-G. Liu S. Liu J.-M. Wang P.-H. (2025). Research on rapid non-destructive detection of tannin and protein content in sorghum based on multi-output Gaussian process. J. Food Composition Anal. 141. doi: 10.1016/j.jfca.2025.107326

[B37] Zhong L. Fan Y. Wu Y. Gao Y. Gao Z. Zhou A. . (2025). Data fusion strategy for rapid prediction of glyceryl trioleate and polysaccharide content in Ganoderma lucidum spore powder based on near-infrared spectroscopy and hyperspectral imaging. J. Food Composition Anal. 141. doi: 10.1016/j.jfca.2025.107403

[B38] Zhu C.-S. Yang C. Feng W.-F. Yuan P.-W. (2025). High-frequency enhanced multi-layer perceptron model fortime series forecasting. J. Comput. Appl., 1–12.

